# Case series of females charged with murder or attempted murder of minors and referred to Weskoppies Hospital in terms of the *Criminal Procedure Act* over a period of 21 years

**DOI:** 10.4102/sajpsychiatry.v22i1.887

**Published:** 2016-05-06

**Authors:** Kwena B. Khoele, Paul H. de Wet, Hermanus W. Pretorius, Jaqui Sommerville

**Affiliations:** 1 South African Military Health Services, South Africa; 2 Department of Psychiatry, Weskoppies Hospital, South Africa; 3 Department of Statistics, University of Pretoria, South Africa

## Abstract

Women charged with murder or attempted murders of children are usually sent for forensic psychiatric evaluation. In South Africa research and literature on this population is scarce. A case series was studied of forensic files of 32 females charged with murder or attempted murder of children. These files contained information on such females. The forensic psychiatric observation was mainly to establish whether a psychiatric diagnosis could be made, and whether they were triable and accountable. Files from 01 Jan 1990 to 31 Dec 2010 (21 years) were obtained of cases observed in Weskoppies Hospital. The aim of describing this case series was to attempt to find a psychiatric profile of such cases, as well as to find other information for example, demographics. The findings, after forensic observation regarding their ability to follow court proceedings and their ability to contribute meaningfully to their defence (trialability) as well as their ability to distinguish between right and wrong, and their ability to act in accordance with the said appreciation (accountability) at the time of the alleged offence were also reported. This information could contribute to making medical practitioners and mental health care workers aware of risk factors involving such cases and to encourage them to enquire about these risk factors.

## Background

It has been reported by Herjanic et al.^1^ that female offenders suffering from ‘affective disorder and neuroses’ [*sic*] were charged with more serious crimes compared to those with schizophrenia or a personality disorder. Herjanic reports findings by Resnick^2^ who found a high incidence of depression among mothers who commit child homicide. Herjanic also reports findings by West^3^ that maternal homicide of small children was the most common type of crime in depressed women. Depression with psychotic features is indicated to be associated with filicide most commonly. The onset of the symptoms is subtle and proceeds unnoticed until florid mood symptoms develop, usually accompanied by psychotic symptoms and utmost aggression.^1^

The Center for Forensic Psychiatry in Ann Arbor, Michigan, studied a sample of 55 women who had murdered their children.^4^ The Center reported that 29 women were psychotic and 26 were not. The psychotic subgroup was older; were more often married or had been married; had a high school or higher level of education; were unemployed; and were not first-time parents. About half of the sample were raised in single-parent families and had been sexually abused. Few of either the psychotic or non-psychotic subjects had a criminal history and none had a history of violent offences.^5^ The most common diagnoses in the psychotic group were schizophrenia and depressive disorder with psychotic features. Personality disorders were also found in this subgroup. The psychotic women had a history of psychiatric hospitalisation and ongoing out-patient treatment. They were reported to have made suicide attempts and had previous homicidal ideation towards their children.^5^ Psychotic women were more likely than non-psychotic women to kill multiple victims. They commonly voiced concerns about their children to family and their psychiatrist less than two weeks before the homicide. About one third of the sample reported severe conflict with their children’s fathers before the crime. Many of the women had multiple, severe stressors. These included financial difficulties, housing problems, ongoing domestic violence, worsening mental illness, limited social support, conflict with family members other than sexual partners, and were primary caregivers for at least one young child. Filicidal mothers typically killed young children (75% of their victims were under six). Children under one were most often victims. Beating and suffocation were the most common methods used by women to kill their children. Multiple researchers have noted the prevalence of serious mental illness, particularly depression and psychosis, among filicidal mothers. This study was among the first involving a relatively large sample of filicidal mothers in the United States of America.^5^

A study by Putkonen et al.^6^ in Finland suggested that neonaticide can be preventable and is more heterogeneous than previously thought. Their sample consisted of 32 women who had concealed their pregnancies prior to their crimes. Among the motives for the concealment of pregnancy were: fear of others’ reactions; fear of abandonment; inability to rear the child; unwanted child; getting rid of baby without anyone’s knowledge; and denial of pregnancy. Motives for the crime were: either unclear or not known; unwanted child; a “panic” situation; fear of abandonment; and inability to cope with the child. Of the 32 subjects, 26 committed the crime in their home, 16 of these in the bathroom. Suffocation was the most common active method followed by strangulation and drowning.^6^

A study carried out by Friedman in Ohio evaluated a large number of factors among 39 mothers who killed their children and were found Not Guilty by Reason of Insanity (NGRI).^7^ The majority of these mothers were married, had a high school education, and some were unemployed primary caregivers in their late 20s. Just over half the children who survived a murder attempt were infants under one year old. The mean age of the children who were killed was preschool age (3–4 years). Almost half of the mothers had previously attempted suicide, and over half (56%) had planned joint filicide-suicides. One half of the mothers had been previously psychiatrically hospitalised and 72% had received prior mental health care. Mothers frequently experienced command hallucinations and delusions about their children. The subjects were often victims of domestic violence, abusive childhoods, or abandonment in childhood. Often, mothers had histories of alcohol- or drug abuse. Though there was a wide range of maternal ages, the mean age of these mentally ill mothers was the late 20s – a similar finding to that of other international studies. Most mothers were, in addition to their mental illness, dealing with stressors, such as single parenting and unemployment. The children were physically healthy, except one. The majority of the mothers had attempted to kill all their children.^7^

A systematic investigation on neonaticide by Spinelli showed that the subjects presented ‘with a childlike demeanour and other features including denial, depersonalization, dissociative hallucinations, and intermittent amnesia at delivery’ [*sic*] and a history of being abused. Some had antisocial personality disorder and high scores on psychiatric tests that suggested malingering. Bizarre actions included returning to bed with the infant’s corpse and keeping it under clothes or in a knapsack.^8^

In the South African context it has been reported that socio-economic stressors play a very important role in the manner in which women with mood disorders and/or adjustment disorders may respond to pregnancy. These conditions can continue or become exacerbated after child birth and/or during the rearing of young children. It was however not possible to assess these factors in this case series as only forensic files were used.

‘Solidarity Helping Hand’ reported shocking statistics regarding South Africa´s current levels of child murder, rape and abuse. During 2007/08, about 1 410 cases of child murder were reported, which was 22% more than the preceding year.^9^ The World Health Organization (WHO) estimated, through the use of limited country-level data, that 53 000 children died worldwide in 2002 as a result of homicide.^10^

## Aim of this case series

The aim of this case series was to describe the psychiatric and personal profiles of women who were charged with murder or attempted murder of children and who were referred to Weskoppies Hospital in Gauteng Province in South Africa for forensic psychiatric observation. Findings regarding the presence of a Mental illness or Mental Defect, if any, their ability to stand trial, and whether they were accountable for their crimes were documented.

The conclusions drawn may help to identify tendencies that might be associated with this type of crime and to establish stressors or other factors that could predispose these women to commit this type of crime.

## Methods

### Study design and case selection

A description of case series of the clinical and administrative files of 32 women charged with a crime of murder and/or attempted murder observed at Weskoppies Psychiatric Hospital between 01 January 1990 and 31 December 2010 (21 years) was undertaken. The clinical characteristics, demographic details, victim details, crime details and observation outcomes were drawn from archived files. The observation outcomes are reported on a standard format containing information regarding: a psychiatric diagnosis (if any); if a mental disorder or mental defect affected the person’s ability to follow court proceedings; if they were able to contribute meaningfully to their defence; and if they were able to distinguish between right and wrong and to act in accordance with the said appreciation. Descriptive statistics were used to summarise and report the results. SAS version 9.2 was used for the analysis.

#### Ethical considerations

Permission to access the information in these files according to the *Promotion of Access to Information Act No 17 of 2000*,^11^ was obtained from the Chief Executive Officer of Weskoppies Hospital. The study was approved by the Research Ethics Committee, Faculty of Health Sciences of the University of Pretoria. Information was accessed from clinical and administrative files of the study population. Each file was assigned a random number. The identity of the accused and/or alleged victims was kept confidential. Such details of the crimes that might lead to identification of the accused or victims were not reported.

## Results

Of the whole group (*n* = 32), in 13 cases no psychiatric diagnosis was made. The subjects with psychiatric diagnoses were grouped into: psychotic illness, mood disorders, substance use disorder, and ‘not mentally ill’ [Tables B]. Tables A contain statistical findings of the whole group. Therefore, in this paper results are presented in five tables of related variables (Tables 1–10). All the subjects who had a mood disorder with psychotic features or who had psychosis and a substance use disorder were included in the ‘psychotic illness’ group. Frequency counts and percentages are expressed in terms of the whole group, and the psychiatric diagnosis subgroups represent only the frequency counts. Most of the results reported focus on the diagnosis subgroups (Part B) instead of the whole sample (Table 1 and Table 2).

**TABLE 1:** Demographic characteristics (*N* = 32).

Group	Variable	All (%)
Age in years	Mean	29.8
	Range	20-49
Married or in a relationship	-	16 (50)
Unemployed	-	24
	-	75
Education	None or	11
	Primary	34
	Secondary	15
	or higher	47
	Unknown	6
	-	19
Where lived	Town	8
	-	25
	Township	14 (44)
	Former	9
	Homeland	28
Lived with husband or partner	-	15 (47)
Religious	-	24 (75)
Biological children	Mean	2.9
	Range	0-8

*Source*: The source is the clinical and administrative files of the study subjects

**TABLE 2:** Demographic characteristics.

Psychotic Illness	Mood Disorders	Substance Use disorder	Not Mentally Ill
9	8	2	13
27.4	32.0	35.5	29.2
22–34	25–39	34–37	20–49
2	6	1	7
8	6	1	9
2	1	1	7
4	6	1	4
1	1	1	5
3	5	0	6
4	2	1	2
2	5	1	7
5	6	2	11
2.4	2.6	4.5	3.1
0–8	1–4	2–7	1–6

*Source*: The source is the clinical and administrative files of the study subjects

The psychotic group had a mean age of 27.4. Seven women (78%) were not married or in a relationship. Two women (22%) lived with their husbands/partners. Four women (44%) had a secondary school or higher levels of education. 8 women (89%) were unemployed. These women had an average of 2.4 biological children (ranges 0–8).

The women with a mood disorder had a mean age of 32. Six women (75%) were married or in a relationship. Five women (62%) lived with their husbands/partners. Six women (75%) had a secondary or higher level of education and six of the women (75%) were unemployed. These women had an average of 2.6 biological children (ranges 1–4).

The two women with a substance use disorder were in their mid-30s. One woman was married and one was employed. One had a secondary or higher level of education. One woman lived with her partner, and these women had an average of 4.5 biological children (ranges 2–7).

The women with no mental illness were between 20 and 49 years (mean age 29.2). Seven (54%) were married or in a relationship, nine (69%) were unemployed and seven (54%) had a primary- or lower level of education. Seven women (54%) were living with their husbands/partners at the time of the crime. The women had an average of 3.1 biological children (ranges 1–6) (Table 3 and Table 4).

**TABLE 3:** Clinical characteristics (*N* = 32).

Group	Variable	All (%)
History of mental illness before the crime	Psychotic	9 (28)
	Mood	2 (6)
	Substance	1 (3)
	MR	2 (6)
	None	19 (59)
Prior psychiatric treatment	Yes	5 (16)
	Unknown	7 (22)
Diagnosis after 30 day observation	Psychotic	9 (28)
	Mood	8 (‘25)
	Substance	2 (6)
	MR	2 (6)
	None	13 (41)
Attempted suicide	-	6 (19)
General medical conditions	-	8 (25)

*Source*: The source is the clinical and administrative files of the study subjects

MR, mental retardation, not documented as an axis 1 diagnosis.

**TABLE 4:** Clinical characteristics.

Psychotic Illness	Mood Disorder	Substance Use Disorder	Not Mentally Ill
9	8	2	13
7	1	0	1
0	2	0	0
0	1	0	0
0	0	1	1
1	4	2	12
3	2	0	0
4	2	0	1
9	0	0	0
0	8	0	0
0	0	2	0
0	0	1	1
0	0	0	13
1	3	0	2
2	3	0	3

*Source*: The source is the clinical and administrative files of the study subjects

MR, mental retardation, not documented as an axis 1 diagnosis.

Nine women (28%) had a diagnosis relating to a psychotic illness. Of note here is that five of these women (55%) had a mood disorder with psychosis. Seven (78%) had a prior history of a psychotic illness and three women (33%) had received prior psychiatric treatment. One woman (11%) with a psychotic illness had attempted suicide and two (22%) had a general medical condition.

Eight women (25%) were diagnosed with a mood disorder. Two of these women (25%) had a mood disorder, one (12, 5%) a psychotic disorder and one (12, 5%) a substance use disorder before the crimes but only two (25%) of the women had received psychiatric treatment. Three women (38%) had attempted suicide and three women (38%) had a general medical condition.

The two women (6%) with a substance use disorder had no history of a mental illness before their crimes. Neither of these women had attempted suicide or had a medical illness. One woman (50%) had mental retardation.

Thirteen women (41%) were found to have no mental illness after observation. One woman (8%) had a history of a psychotic illness before the crime. Two (15%) had attempted suicide and three (23%) had a medical illness, and one (8%) had mental retardation (Table 5 and Table 6).

**TABLE 5:** Victim and crime details (*N* = 32).

Group	Variable	All (%)
Age	0–24 hours	5 (12)
	1 day-1 year	17 (42)
	2–15 years	18 (45)
Gender	Male	24 (60)
	Female	14 (35)
Relationship of accused	Mother	37 (92)
Order in family	First born	10 (25)
	Second	15 (38)
Crime method	Weapon	10 (25)
	Poisoning	8 (20)
	Drowning	4 (10)
Crime location	Home	33 (82)
Body location	Home	14 (35)
	Toilet	4 (10)

*Source*: The source is the clinical and administrative files of the study subjects

Note: There were multiple victims in 7 of the 32 cases. Frequency counts and percentages of the victims are expressed in terms of the 40 victims, not the 32 women.

**TABLE 6:** Victim and crime details.

Psychotic Illness	Mood Disorder	Substance Use Disorder	Not Mentally Ill
9	8	2	13
10	10	3	17
1	1	1	2
6	4	0	7
3	5	2	8
6	7	3	8
3	2	0	9
8	10	3	16
1	3	1	5
5	4	1	5
4	1	0	5
0	5	0	3
3	1	0	0
7	9	3	14
5	3	2	4
1	1	1	1

Six victims (60%) of the women in the psychotic group were between 1 day and 1 year old. Six of the victims (60%) were male and eight women (80%) were the mothers of the victims. Second-born children (5; 50%) were commonly killed. Weapons were the most commonly used method (4; 40%). The most common crime location was the home (7; 70%) and the victims’ bodies were also most commonly found inside their homes (5; 50%).

Five victims of the women with a mood disorder were in the age group 2 to 15 years (50%); seven victims were male (70%) and one child`s gender was not recorded. All of the women were mothers to the victims. Second-born children were the most common victims (4; 40%) and poisoning was used in more cases (5; 50%) than any other method. The crimes were committed in their homes (9; 90%). The victims’ bodies (3; 30%) were found at their homes.

Two of the three (67%) victims of the women with a substance use disorder were in the age group 2 to 15 years and all the three victims were males. One woman (33%) had mental retardation. All the victims were the women’s biological children. First- and second-born children were equally involved (1 each) and one victim’s order in the family was not recorded. All the crimes were committed at home and the victims’ bodies were found at home (2; 67%) or in the toilet (1; 33%) [Two victims did not die].

Seven victims (41%) of the women with no mental illness were aged between one day and one year old and eight (47%) between 2–15 years old. Nine victims (53%) were female. The women were mothers of 16 out of the 17 victims. First- and second-born children were equally involved (both 5; 29%). Weapons were commonly used in the crimes (5; 29%) and fourteen (82%) of the crimes were committed at home. The bodies were found at their homes (4; 24%) or in the toilet outside the home (1; 6%) (Table 7 and Table 8).

**TABLE 7:** Forensic details (*N* = 32).

Group		All (%)
Charge**	Murder	22
	-	69
	Attempted	10
	murder	31
Single victim	-	25
	-	62
Reason expressed	-	23
	-	72
Reason	Relationship	8
	problems with partners	25
	Conflict within subject´s family***	8
	-	25

*Source*: The source is the clinical and administrative files of the study subjects

** Three women were accused of both murder and attempted murder, which explains why there is a total of 16 women in the ‘no mental illness’ group (Table IV: B).

*** Conflict within subject’s family includes family members other than the partner.

**TABLE 8:** Forensic details.

Psychotic Illness	Mood Disorder	Substance Use Disorder	Not Mentally Ill
9	8	2	13
8	5	1	8
1	3	1	2
8	6	1	10
6	7	2	8
1	2	1	4
2	2	0	4

*Source*: The source is the clinical and administrative files of the study subjects

**, Three women were accused of both murder and attempted murder, which explains why there is a total of 16 women in the “no mental illness” group (Table IV: B).

***, Conflict within subject’s family includes family members other than the partner.

Eight women (89%) in the psychotic group were charged with murder and eight women (89%) had a single victim. Of these, six women (67%) expressed a reason for their crimes, and the most common reason given was family conflict.

Five women (62%) with a mood disorder were charged with murder and three (38%) were charged with attempted murder. Six women (75%) had a single victim. Seven of these women (88%) expressed a reason for the crimes, which were mostly stated as relationship problems (2; 25%) and family conflict (2; 25%).

One woman (50%) in the substance use disorder group was charged with murder and one (50%) with attempted murder. One had a single victim and the other woman had two victims. Relationship problems were given as a reason by one of these women.

Eight women (61%) with no mental illness were charged with murder, two (15%) with attempted murder, and three (23%) were charged with both murder and attempted murder. Three of the crimes (33%) involved more than one victim. The reasons given were relationship problems (4; 31%) and family conflict (4; 31%) (Table 9 and Table 10).

**TABLE 9:** Observation Outcomes (*N* = 32).

Group	All (%)
Trialable	22 (69)
Accountable	18 (56)

*Source*: The source is the clinical and administrative files of the study subjects

**TABLE 10:** Observation outcomes.

Psychotic Illness	Mood Disorder	Substance Use Disorder	Not Mentally Ill
9	8	2	13
2	5	2	13
0	3	2	13

*Source*: The source is the clinical and administrative files of the study subjects

Two women (22%) with a psychotic illness were found to be triable for their crimes and none of them were found accountable. Five women (62%) with a mood disorder were triable and three of them (38%) were found accountable. The two women (100%) with substance use disorder were triable and accountable for their crimes. Thirteen women (100%) with no mental illness were triable and accountable for their crimes. The overall findings shows that 22 women (69%) of the offenders were found to be triable in court and 18 women (56%) were found to be accountable.

## Discussion

These 32 case series show that a high percentage (41%) of the subjects had no mental illness, followed by those with psychotic illness (28%), mood disorders (25%) and substance use disorders (6%). None of the women were diagnosed with a personality disorder. Only six of the subjects in this case series had attempted suicide. The victims were commonly male and most of the subjects were the victim`s mother with more than one biological child. All the subjects with substance use disorders and those with no mental illness were found to be suitable to stand trial. The latter subjects were also found to be accountable for their crimes compared to a lower incidence in the psychotic and mood disorder groups.

The mean age of the psychotic group was late 20s and they were not married or in a relationship, many had a high school education or higher and they were commonly unemployed, similar to the Friedman study.^7^ A higher percentage of the children killed by the psychotic women were between one day and one year old in contrast to the Friedman study, which found the victims to be of preschool age (3–4 years). Most of the women in our case series had a history of psychosis before the crime but only 33% were reported to have received psychiatric care, while international studies report that a high percentage of subjects had received prior psychiatric care and had voiced concerns about their children to family and their psychiatrist before the homicide.^4,5,6,7^ Most of the women in these internationally reported studies had experienced command hallucinations and delusions against their children, domestic violence, abusive childhoods, financial difficulties, worsening mental illness, conflict with sexual partners and histories of alcohol or drug abuse.^4,5,7^ A high number of the women in our case series reported relationship problems and family conflict as a reason for their crimes and they commonly used weapons and poisoning to kill their children.

International reviews show a high frequency of depression, suicidal behaviour and malingering among mothers who commit child homicide. Most of the women in our psychotic group (55%) had a mood disorder with psychotic features and an additional eight women had a mood disorder without psychosis. This confirms a high incidence of mood disorders among the women diagnosed with a mental illness in our case series. The mean age of these women with mood disorders was early 30s and most were married or in a relationship, unemployed, and had a secondary- or higher level of education. Their victims were commonly between 2 and 15 years old. They commonly poisoned their victims and most of them had only one victim. Few of these women had suicidal behaviour before committing the crime and malingering was not diagnosed.

In 13 of our cases the victims’ ages were between 0 day to 1 year and the mothers were found to be mentally ill (high percentage of psychosis and mood disorders), which could indicate the possibility of post-partum mental illness. This finding is in agreement with international studies reporting a high frequency of depressive and psychotic disorders in filicidal mothers.^4,5,12,13,14^ Post-partum depression is often associated with bipolar illness and postpartum psychiatric illness is clearly a treatable risk factor for filicide. If postpartum depression or psychosis is diagnosed, the safety of the mother and her children should first be assured with admission and intensive psychiatric management of the mother. Possible temporary placement of children with an alternative caregiver should be considered. It is important for clinicians to monitor the postpartum depressed woman closely because this may indicate the depressive phase of bipolar disorder. Differentiation of bipolar depression is mandatory because pharmacotherapy for unipolar depression can induce a manic switch, cycle acceleration, and even suicide risk.^4^

### Study limitations

This case series has a few limitations. Firstly, a small sample did not allow an elaborate analysis of the problem. Secondly, the women assessed were only those referred by the court for observation so the results cannot be generalised to all filicidal women. Thirdly, the study population had not necessarily received prior care from Weskoppies Hospital so their psychiatric histories could not be confirmed.

Although it has been pointed out that socio-economic stressors play a role as far as how mood disorders and adjustment disorders may respond to pregnancy and child rearing, it was not possible to assess these factors in our case series as only forensic files were used.

## Conclusion

These case series show psychotic illness, mood disorders and an unconfirmed possibility of postpartum illness as possible factors that predisposed the women studied to commit child homicide (6 out of 10 victims of mothers in the psychotic subgroup and 4 out of 10 victims of mothers in the mood disorder group were between one day and one year old). Relationship problems and family conflict were identified as common reasons for the crimes. A high number of the women without psychosis and mood disorders were triable and accountable for the crimes.

The fact that the vast majority of child homicides are carried out by women with no previous criminal record suggests that this phenomenon is not about some deranged, evil woman killing her children in cold blood. Rather, there is a context behind such a desperate act and it is important to understand that context, so that effective interventions can be implemented.^13^

Little is known about the prevention of filicide committed by mentally ill mothers, and there is a dearth of research focused on identifying specific risk factors. Research has not comprehensively described the profiles of mentally ill mothers who kill their children.^7^

Research to explore the character and quality of mental illness associated with filicide is important because approximately one fourth of the women referred to psychiatric services had a child under the age of 5 years.^4^ All medical practitioners (especially psychiatrists, mental health care workers and obstetricians) should enquire about filicidal/homicidal thoughts when following up mothers with children who are either inpatients or outpatients. Mental health care workers should specifically not allow pregnant mothers and/or mothers with small children to default treatment, as this could possibly be a preventative measure.

Recent statistics reveal that the rate of child murder in South Africa has increased by 22% for the financial year of 2007/2008, bringing the total number of children murdered to 1410 (SA Government Information, 2008). How many of these murders are committed by the biological mothers and/or fathers of these children and how are these murders different or similar to parental child homicide committed in other parts of the world?^13^ This review found a high incidence of mood disorders but few had suicidal behaviour before the incident, while international reviews found a high frequency of depression, suicidal behaviour and malingering before the incident.

Child murder appears to be a multifaceted phenomenon that deserves a high level of professional attention to identify families that are likely to be prone to this kind of tragedy. In this way the appropriate intervention strategies can be developed. Prevention implies the recognition of risk factors involved in each situation and the identification of potential subjects. The possibility of homicidal tendencies should never be underestimated in depressive subjects, particularly those of mothers. Therefore, medical doctors do indeed have a significant role to play in the prevention of child murder.^5^
